# The Promoting Effect of *Ishige sinicola* on Hair Growth

**DOI:** 10.3390/md11061783

**Published:** 2013-05-24

**Authors:** Jung-Il Kang, Eun-JI Kim, Min-Kyoung Kim, You-Jin Jeon, Sung-Myung Kang, Young-Sang Koh, Eun-Sook Yoo, Hee-Kyoung Kang

**Affiliations:** 1 Department of Medicine, School of Medicine, Institute of Medical Sciences, Jeju National University, 102 Jejudaehakno, Jeju 690-756, Korea; E-Mails: asdkji@hanmail.net (J.-I.K.); ejk8730@naver.com (E.-J.K.); loveis6776@hanmail.net (M.-K.K.); yskoh7@jejunu.ac.kr (Y.-S.K.); eunsyoo@jejunu.ac.kr (E.-S.Y.); 2 Aqua Green Technology Co. Ltd., 209 Jeju Bio-Industry Center, 102 Jejudaehakno, Jeju 690-121, Korea; E-Mail: youjinj@jejunu.ac.kr; 3 Department of Marine Life Science, Jeju National University, 102 Jejudaehakno, Jeju 690-756, Korea; E-Mail: tjdaud81@hanmail.net

**Keywords:** *Ishige sinicola*, hair growth, dermal papilla cells, β-catenin, 5α-reductase

## Abstract

This study was conducted to evaluate the promoting effect of *Ishige sinicola*, an alga native to Jeju Island, Korea, on hair growth. When vibrissa follicles were cultured in the presence of *I. sinicola* extract for 21 days, *I. sinicola* extract increased hair-fiber length. After topical application of *I. sinicola* extract onto the back of C57BL/6 mice, anagen progression of the hair shaft was induced. The *I. sinicola* extract significantly inhibited the activity of 5α-reductase. Treatment of immortalized vibrissa dermal papilla cells (DPCs) with *I. sinicola* extract resulted in increase of cell proliferation, which was accompanied by the increase of phospho-GSK3β level, β-catenin, Cyclin E and CDK2, whereas p27^kip1^ was down-regulated. In particular, octaphlorethol A, an isolated component from the *I. sinicola* extract, inhibited the activity of 5α-reductase and increased the proliferation of DPCs. These results suggest that *I. sinicola* extract and octaphlorethol A, a principal of *I. sinicola*, have the potential to treat alopecia via the proliferation of DPCs followed by the activation of β-catenin pathway, and the 5α-reductase inhibition.

## 1. Introduction

Recently, there have been an increasing number of people suffering from hair loss, which has mostly emerged from psychological and physical stress [1]. Nevertheless, the underlying causes of baldness are poorly understood. Many materials have been used to cure alopecia. However, only two drugs so far have been approved for hair loss treatment by the Food and Drug Administration (FDA, Rockville, MD, USA); finasteride and minoxidil [2,3]. Finasteride, a type II 5α-reductase inhibitor, was initially used for curing prostatic hypertrophy [4], but later found to stimulate hair growth in men with androgenetic alopecia (AGA), which is the most common type of alopecia in men over the age of 40 [4,5,6]. Minoxidil, a potassium channel opener was developed as an anti-hypertensive. Moreover, it was also found to stimulate hair growth by the opening of ATP-sensitive K^+^-channel [7,8], the up-regulation of vascular endothelial growth factor (VEGF) [9] and the activation of β-catenin pathway [10] in dermal papilla cells (DPCs). The DPCs are mesenchymally-derived cells which play important roles in the morphogenesis, regeneration, and growth of hair [11]. Han *et al.* reported that minoxidil has proliferative and anti-apoptotic effects on DPCs [12]. Several natural products were also reported to promote hair growth via the proliferation of DPCs [13,14,15]. Wnt/β-catenin plays an important role in hair growth, regeneration as well as cell proliferation [16,17,18]. β-Catenin is a main component of the Wnt pathway, and the level of β-catenin is regulated by degradation complexes such as adenomatous polyposis coli (APC), glycogen synthase kinase-3β (GSK-3β), axin, and casein kinase I. Accumulation of nuclear β-catenin results in the activation of target genes such as cyclin D1 and c-myc [19,20]. Cell cycle regulation is a crucial event for cell proliferation and is tightly regulated by cyclin/cyclin-dependent kinases (CDKs) and CDK inhibitors [21]. Migogen-induced cell cycle progression can be inhibited by p27^kip1^, a CDK inhibitor, which contributes to inhibit cell proliferation [21,22].

To develop new therapies to enhance hair growth, we screened the extracts of Jeju algae and discovered that *Ishige sinicola* has the potential to promote hair growth. *I. sinicola*, a brown alga, was reported to have anti-bacterial and anti-inflammatory effects against acne [23]. Octaphlorethol A, a component from *I. sinicola*, was reported to induce anti-diabetic activity in skeletal muscle [24]. However, the effect of *I. sinicola* and the bio-active components of *I. sinicola* on the promotion of hair growth have not yet been reported. Therefore, the present study was carried out to investigate the promoting effect of *I. sinicola* extract and as an active component regarding hair growth.

## 2. Results

### 2.1. The Effect of *I. sinicola* Extract on the Hair-Fiber Elongation of Rat Vibrissa Follicle

**Figure 1:**
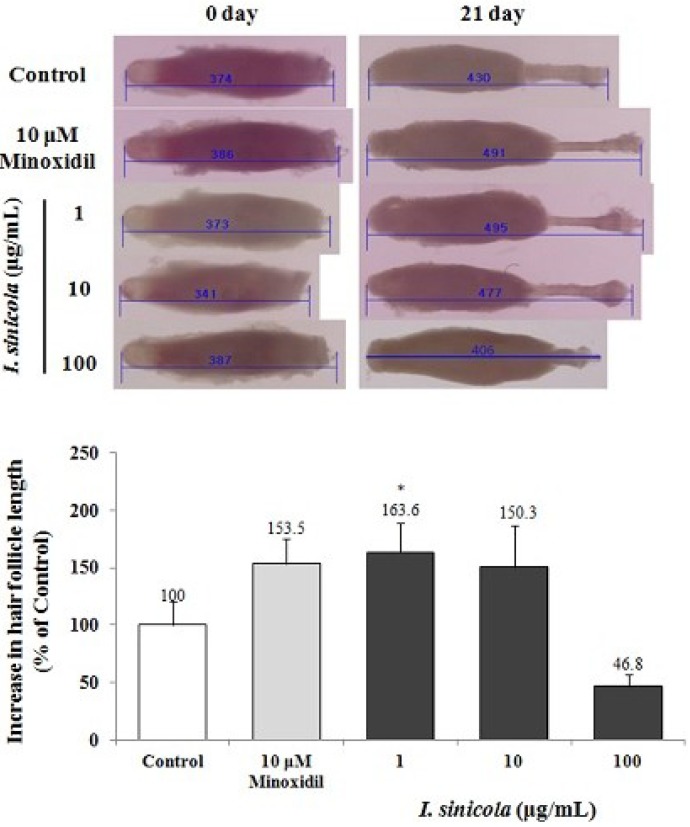
The elongation effect of *I. sinicola* extract on the hair-fiber length of rat vibrissa follicle. Individual vibrissa follicles from Wistar rats were microdissected and then cultured in William’s E medium at 37 °C under 5% CO_2_. Vibrissa follicles were then treated with *I. sinicola* extract (1, 10 and 100 μg/mL) for 21 days. Stimulation with minoxidil served as a positive control. All experiments were performed in triplicate. The difference in the length of vibrissa follicles of the vehicle-treated control group on day 21 was taken to be 100%. Data are presented as the percentage of the length of the treated follicles based on the mean length of the control follicles ± the S.E. * p < 0.05 *vs.* control.

To determine whether *I. sinicola* extract could promote hair growth, we examined the effect of *I. sinicola* extract using an organ culture of the rat vibrissa follicle. When rat vibrissa follicles were treated with various concentrations of *I. sinicola* extract for 3 weeks, in particular, the hair-fiber length of the vibrissa follicles treated with 1 μg/mL of *I. sinicola* extract significantly increased compared with the control (Figure 1). However, 100 μg/mL of *I. sinicola* extract decreased the hair-fiber length compared with the control.

### 2.2. The Effect of *I. sinicola* Extract on the Anagen Induction in C57BL/6 Mice

**Figure 2:**
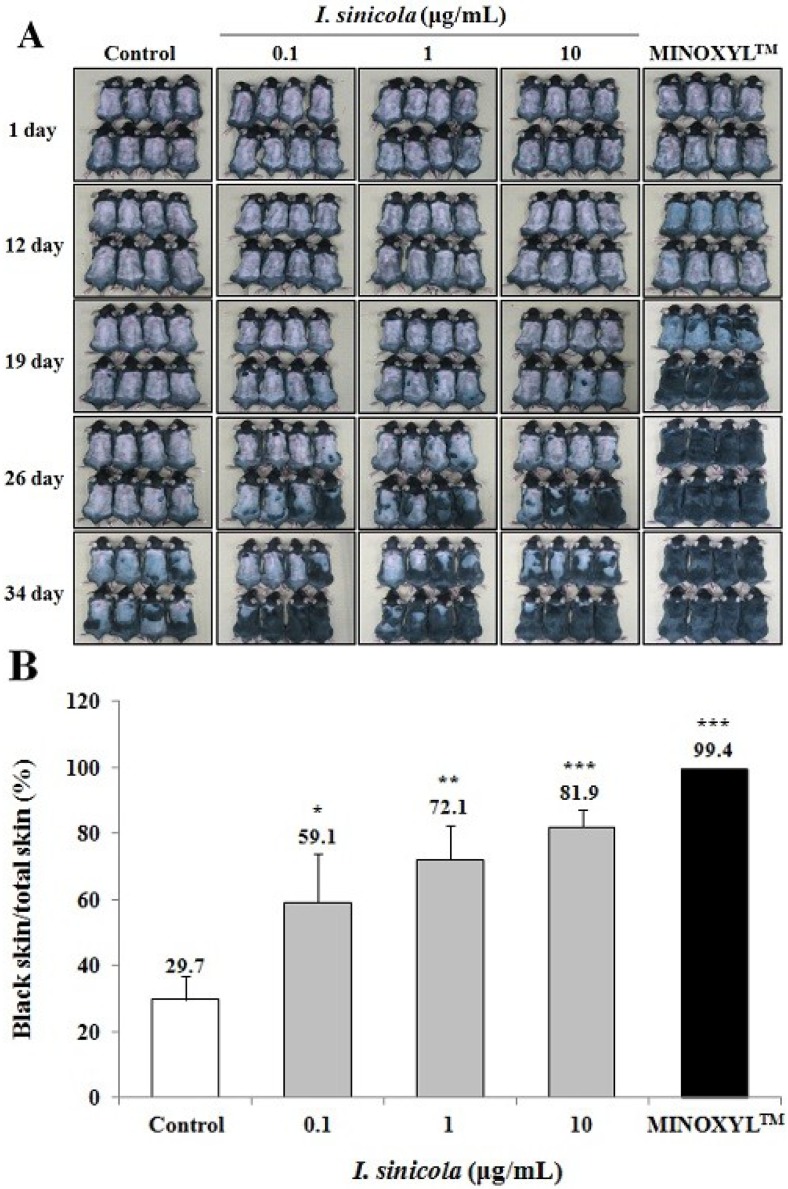
The effect of *I. sinicola* extract on the anagen induction in C57BL/6 mice. After shaving, the back skins were treated with *I. sinicola* extract, vehicle and 5% minoxidil (MINOXYL™) every day for 34 days. (**A**) The back skins were photographed at 1, 12, 19, 26 and 34 days after depilation; (**B**) On day 34, to analyze the quantitative assessment of anagen induction, dotmatrix planimetry was performed. The transparency was put on a photo of a mouse to mark the areas that were in different stages (pink = telogen, anagen = black). Afterward a dotmatrix (sheet with a uniform defined dot pattern) was placed under the marked foil to calculate the percentages of the regions of interest by counting the dots. The percentage of anagen induction was calculated by the equation [(black skin/total skin) × 100]. Data are presented as the mean ± S.E. (*n =* *8*). * p < 0.05, ** p < 0.01, *** p < 0.001 *vs.* vehicle treated control.

To investigate whether anagen induction was promoted by *I. sinicola* extract, we used C57BL/6 mice, since the dorsal hair is known to have a time-synchronized hair growth cycle [25]. Shaved skin of telogen C57BL/6 mice is pink color, which then darkens along with anagen initiation. As shown in Figure 2, 10 μg/mL of *I. sinicola* extract-treated group showed gray skin at 19 days after depilation. When the area of black skin was analyzed with dotmatrix planimetry, the black skin area of the *I. sinicola* extract treated group was significantly larger than that of the control group at 34 days after depilation. The 5% Minoxidil (Minoxidil™) treated group, a positive control group, exhibited gray skin from 12 days after depilation. These results indicate that *I. sinicola* extract promoted telogen-to-anagen transition in C57BL/6 mice.

### 2.3. Effects of *I. sinicola* Extract on the 5α-Reductase Activity and the Proliferation of DPCs

**Figure 3:**
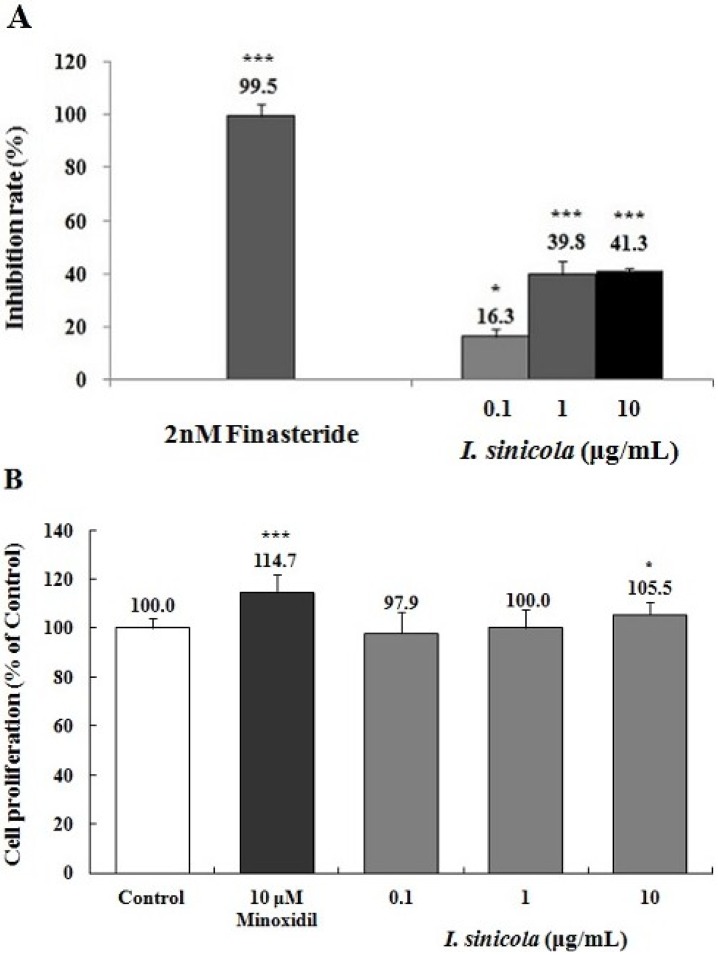
The effects of *I. sinicola* extract on the inhibition of 5α-reductase and the proliferation of dermal papilla cells (DPCs). (**A**) Assay of 5α-reductase inhibition was performed using crude extract of rat prostate as described in “Materials and Methods”. The conversion rate of testosterone (T) to dihydrotestosterone (DHT) was calculated by the equation: [DHT/(T + DHT)] × 100. Inhibition rate (%) was expressed as a percentage of reduced conversion rate compared to the control. The inhibition activity of control group was regarded as 0% (not shown). Finasteride was used as a positive control. (**B**) Immortalized vibrissa DPCs (1.0 × 10^4^ cells/mL) were plated in 96 well plates. DPCs were treated with various concentration of *I. sinicola* extract, as indicated. Cell proliferation was measured using a MTT assay for 4 days. Minoxidil (10 μM) was used as a positive control. Data are presented as the mean ± the S.D. of three independent experiments. * p < 0.05, ** p < 0.01, *** p < 0.001 *vs.* control.

5α-Reductase is an enzyme involved in conversion of testosterone to dihydrotestosterone (DHT) and a potential target of prevention of hair loss [26,27]. To determine whether *I. sinicola* extract could inhibit 5α-reductase activity, we examined the 5α-reductase activity with crude enzyme from rat prostate. As shown in Figure 3A, *I. sinicola* extract inhibited 5α-reductase activities by 16.3%, 39.8% and 41.3% at the concentration of 0.1, 1 and 10 μg/mL, respectively. Finasteride, a positive control, inhibited 5α-reductase activity by above 84.5% at 2 nM concentration. The results suggest that *I. sinicola* extract could have the potential for the treatment of AGA via the 5α-reductase inhibition. 

To evaluate the effect of *I. sinicola* extract on cell proliferation of hair follicles, DPCs were treated with various concentrations of *I. sinicola* extract. The *I. sinicola* extract promoted the proliferation of DPCs by 105.5% at the concentration of 10 μg/mL compared with the vehicle-treated control (Figure 3B). However, 100 μg/mL of *I. sinicola* extract inhibited the proliferation of dermal papilla cells compared to the control group (data not shown). The inhibition of DPC proliferation seems to result in the decrease of hair-fiber length (Figure 1). Minoxidil, a positive control, enhanced the proliferation of DPCs by 114.7% at the concentration of 10 μM compared with the vehicle-treated control. The results suggest that *I. sinicola* extract might have hair-growth promoting effect via the proliferation of DPCs.

### 2.4. Effects of *I. sinicola* Extract on the Expression Levels of Cell Cycle-Associated Proteins and β-Catenin

**Figure 4:**
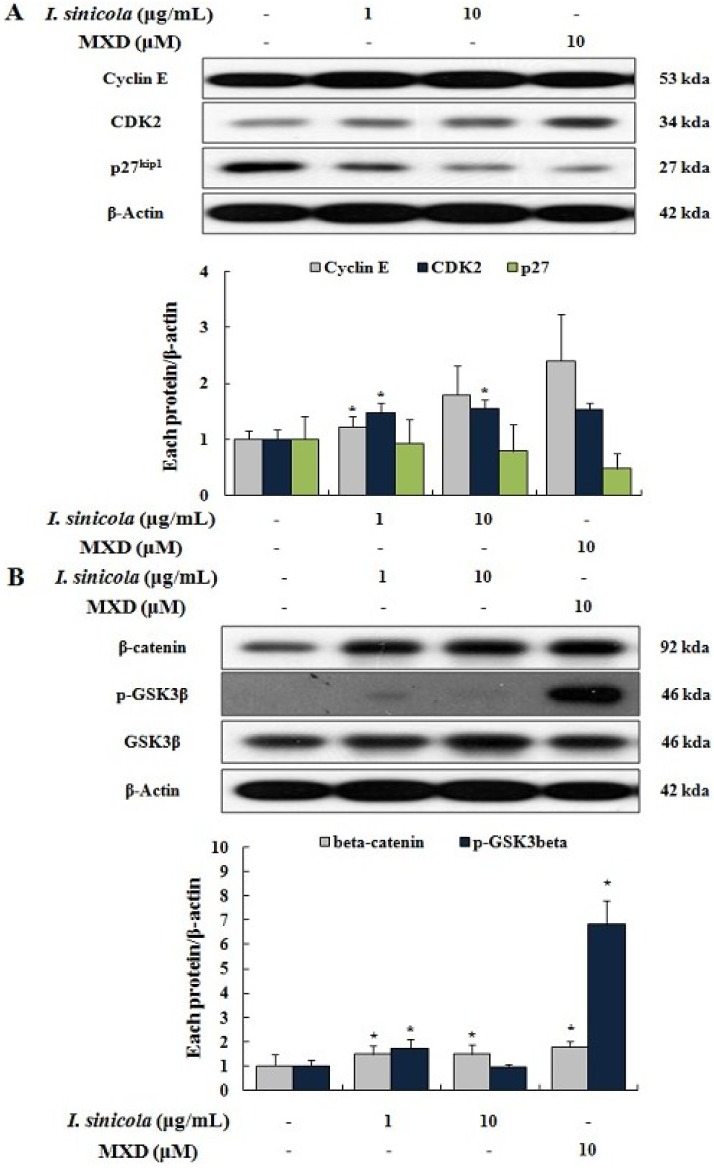
The *I. sinicola* extract regulates the levels of cell cycle associated proteins and the Wnt/β-catenin signaling proteins in cultured DPCs. Immortalized vibrissa DPCs (1.0 × 10^5^ cells/mL in 100 mm dishes) were pre-incubated for 24 h under 1% serum conditions, the cells were treated with *I. sinicola* extract (1 and 10 μg/mL) and minoxidil (MXD, 10 μM) as a positive control for 24 h. Whole cell lysates from DPCs were analyzed for (**A**) the levels of Cyclin E, CDK2 and p27^kip1^ as well as (**B**) the levels of phospho-GSK3β, GSK3β and β-catenin by western blot. Lower panel displays mean ± S.E. from three independent experiments. * p < 0.05 *vs.* control.

Mammalian cells require activation of Cyclin E/CDK2 for cell cycle progression which is key process for cell proliferation [28]. Among cell cycle-associated proteins, p27^kip1^ binds to Cyclin E/CDK2 complexes and inhibits activation of Cyclin E/CDK2 [22,28]. In order to determine whether the proliferative effect of *I. sinicola* extract mediated by regulation of cell cycle proteins, we examined the expressions of p27^kip1^, Cyclin E and CDK2 (*p <* 0.05). *I. sinicola* extract decreased the expression of p27^kip1^, while increased the expressions of Cyclin E and CDK2 (Figure 4A). When treated with minoxidil (10 μM), similar results were obtained (Figure 4A). On the other hand, Wnt/β-catenin pathway plays an important role in hair growth [29], and has been implicated in the regulation of cell cycle proteins [19,30]. Activation of Wnt signaling pathway by phosphorylation of GSK3β induces the stabilization of β-catenin, which can cause nuclear translocation of β-catenin [31]. As shown in Figure 4B, when treated with *I. sinicola* extract, the level of β-catenin and the phosphorylation of GSK3β were increased. Minoxidil also increased the levels of β-catenin and the phospho-GSK3β (*p <* 0.05), compared to the control cells (Figure 4B). These results suggest that *I. sinicola* induced proliferation of DPCs via the regulation of cell cycle and Wnt/β-catenin signaling.

### 2.5. Effect of Octaphlorethol A on the 5α-Reductase Activity and the Proliferation of DPCs

**Figure 5:**
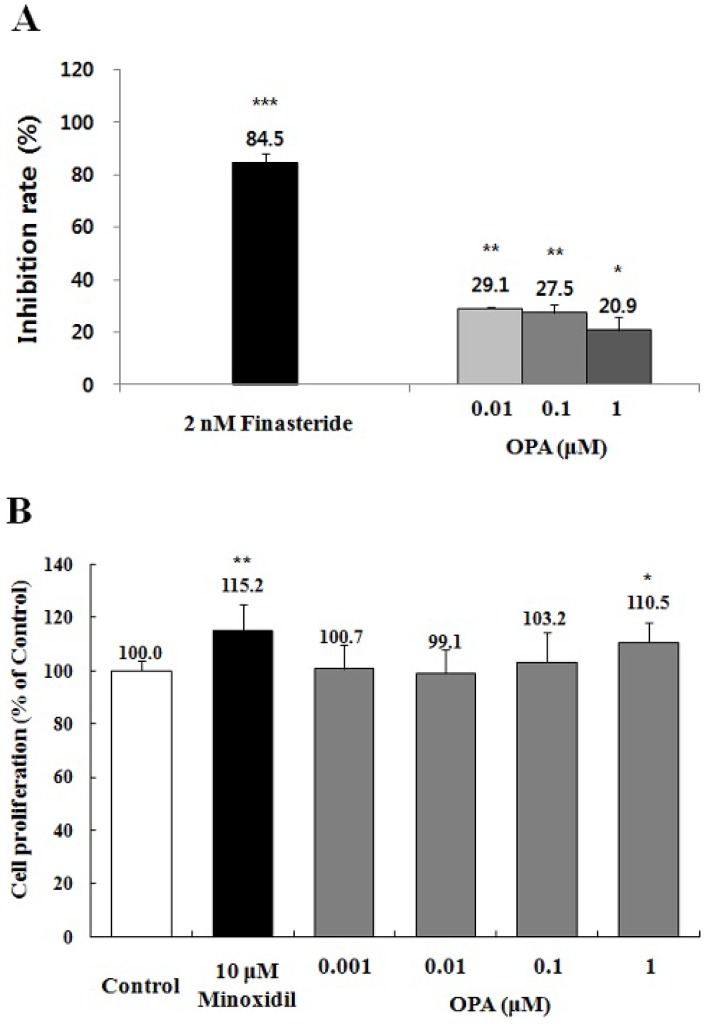
The effects of octaphlorethol A (OPA) on the inhibition of 5α-reductase and the proliferation of DPCs. (**A**) Assay of 5α-reductase inhibition was performed using crude extract of rat prostate as described in “Materials and Methods”. The conversion rate of testosterone (T) to dihydrotestosterone (DHT) was calculated by the equation [DHT/(T + DHT)] × 100. Inhibition rate (%) was expressed as a percentage of reduced conversion rate compared to the control. The inhibition activity of control group was regarded as 0% (not shown). Finasteride was used as a positive control; (**B**) Immortalized vibrissa DPCs (1.0 × 10^4^ cells/mL) were plated in 96 well plates. DPCs were treated with various concentration of OPA, as indicated. Cell proliferation was measured using a MTT assay for 4 days. Minoxidil (10 μM) was used as a positive control. Data are presented as the mean ± the S.D. of three independent experiments. * p < 0.05, ** p < 0.01, *** p < 0.001 *vs.* control.

To search the active components from *I. sinicola* extract on the promotion of hair growth, firstly, octaphlorethol A, a known component of *I. sinicola*, was examined. To evaluate whether octaphlorethol A could inhibit 5α-reductase activity, we examined the 5α-reductase activity with crude enzyme from rat prostate. Octaphlorethol A inhibited 5α-reductase activities by 29.1%, 27.5% and 20.9% at the concentration of 0.01, 0.1 and 1 μM, respectively (Figure 5A). Finasteride, a positive control, inhibited 5α-reductase activities by above 84.5% at 2 nM concentration. The results suggest that octaphlorethol A could have the potential for the treatment of AGA via the 5α-reductase inhibition.

We also examined whether octaphlorethol A can also promote the proliferation of DPCs. Octaphlorethol A increased the proliferation of DPCs by 103 and 110.5% at the concentration of 0.1 and 1 μM compared with the vehicle-treated control (Figure 5B). The results suggest that octaphlorethol A might have a hair-growth promoting effect via the proliferation of DPCs.

## 3. Discussion

In this study, the hair-growth promoting effects of *I. sinicola* extract and octaphlorethol A, a principal of *I. sinicola* extract, were investigated. To the best of our knowledge, this study is the first to demonstrate that *I. sinicola* extract and octaphlorethol A have the potential to treat alopecia via the proliferation of DPCs and 5α-reductase inhibition.

To investigate hair-fiber elongation, we adopted rat vibrissa follicle culture model [32]. Vibrissa follicles from rats are much larger than pelage follicles and can be successfully cultured *in vitro* [33]. In particular, the hair growth cycles of the rat vibrissa follicles have been reported to be synchronized according to their age [33]. In addition, the isolated rat vibrissa follicles can be maintained *in vitro* up to 23 days [33]. In this study, we have isolated large posterior vibrissa follicles from 23 days old rats and maintained them for up to 21 days *in vitro*. In the continuing search for hair-growth promoting agents from natural sources, we found that the *I. sinicola* extract promoted the elongation of hair-fiber in cultured rat vibrissa follicles (Figure 1). To evaluate the *in vivo* effect of *I. sinicola* extract on the induction of the anagen phase, the hair growth promoting effect on C57BL/6 mouse was examined. As shown in Figure 2, *I. sinicola* extract promoted the anagen initiation of C57BL/6 mice in a dose-dependent manner.

AGA, the most common type of alopecia, can be modulated by the inhibition of 5α-reductase, which converts testosterone to DHT [2,4,34]. Finasteride is known to repress the progression of AGA through inhibition of 5α-reductase [1,34]. We found that *I. sinicola* extract and octaphlorethol A could inhibit the activity of 5α-reductase (Figure 3, Figure 5). The results mean that *I. sinicola* extract and octaphlorethol A could have the potential for the treatment of AGA via the 5α-reductase inhibition.

The mesenchymally-derived dermal papilla cells play a pivotal role in hair growth regulation. The morphology of dermal papilla cells can be altered through the hair growth cycle, being maximal in volume in the growing phase (anagen) and least in the resting phase (telogen). Evidence has shown that the size of dermal papilla cells is well correlated with hair growth, and the cell number of dermal papilla cells is increased in the growing phase of hair cycle [35,36]. *I. sinicola* extract and octaphlorethol A increased the proliferation of DPCs compared with the control group (Figure 3, Figure 5). The results indicate that *I. sinicola* extract and octaphlorethol A might have hair-growth promoting effect via the proliferation of DPCs.

Progression of cell cycle is important event in the cell proliferation and is driven by cycle/CDKs complexes and CDK inhibitors [37,38]. As shown in Figure 4A, *I. sinicola* extract increased the expressions of Cyclin E and CDK2, whereas the expression of p27^kip1^ decreased. Minoxidil treatment also showed similar results (Figure 4). These results suggest that *I. sinicola* extract and minoxidil increased the proliferation of DPCs via the regulation of cell cycle progression. Glibenclamide, a K_ATP_ channel blocker, induced the cell cycle and inhibited cell proliferation by minoxidil, a K_ATP_ channel opener [39]. Minoxidil is also known to induce hair growth through the activation of Wnt/β-catenin pathway which is a known regulator of hair growth [10,16,17]. Our group also previously reported that acankoreoside J from *Acanthopanax koreanum* could increase hair growth through cell cycle progression and nuclear location of β-catenin [14]. The increase of GSK3β has been known to induce the activation and nuclear translocation of β-catenin [31]. In the present study, *I. sinicola* extract increased the levels of phospho-GSK3β and β-catenin (Figure 4B). These results are similar to other studies in which DPCs proliferation can be promoted by minoxidil or GSK3β inhibitor [10,12,40]. Therefore, our results indicate that proliferation of DPCs by *I. sinicola* extract can be promoted through the increase of Cyclin E, CDK2, β-catenin and phosph-GSK3β, and the down-regulation of p27^kip1^.

In the present study, we demonstrated that *I. sinicola* extract could promote hair growth *in vitro* and *in vivo.* Furthermore, *I. sinicola* extract increased the proliferation of DPCs via the up-regulation of Cyclin E, CDK2, β-catenin and phosph-GSK3β, and the down-regulation of p27^kip1^. On the other hand, *I. sinicola* extract significantly inhibited the 5α-reductase activity. In the course of searching the components promoting hair-growth, we found that octaphlorethol A, a known compound of *I. sinicola*, could increase the proliferation of DPCs and inhibit the 5α-reductase activity. Our results indicate that octaphlorethol A can show both beneficial characteristics of minoxidil and finateride in the treatment of alopecia. The results suggest that *I. sinicola* extract and octaphlorethol A, a principal of *I. sinicola*, might help to treat alopecia via the proliferation of DPCs followed by the activation of β-catenin pathway, and the 5α-reductase inhibition.

## 4. Experimental Section

### 4.1. The Preparation of *I. sinicola* Extract and Isolation of Octaphlorethol A

*Ishige sinicola*, a brown alga, was collected along the coast of Jeju Island, Korea, between Jun and July, 2010. The seaweed was washed three times with tap water to remove the salt, epiphytes, and sand attached to the surface, then carefully rinsed with fresh water and maintained in a medical refrigerator at −20 °C. Thereafter, the frozen sample was lyophilized and homogenized with a grinder prior to extraction. The powder (500 g) was extracted with 80% aqueous ethanol (12 L) at room temperature for 24 h and filtrated. After filtration, the 80% ethanol extract was evaporated to dryness under vacuum and used as *I. sinicola* extract. Octaphlorethol A was isolated according to methods described in previous report [24]. The *I. sinicola* extract and octaphlorethol A were dissolved in dimethyl sulfoxide (DMSO) (Sigma, St. Louise, MO, USA) for subsequent treatment adjusting the final concentration of DMSO in culture medium to <0.2%. 

### 4.2. Animals

Three-Week-old male Wistar rats, 6-week-old female C57BL/6 mice and 8-week-old male spargue-Dawley (SD) rats were supplied from Orient Bio (Seongnam, Gyeonggi, Korea). All animals provided with a standard laboratory diet and water *ad libitum*. All animals were cared for by using protocols (20100031) approved by the Institutional Animal Care and Use Committee (IACUC) of the Jeju National University. 

### 4.3. Isolation and Culture of Rat Vibrissa Follicles

Isolation of rat vibrissa follicles was performed as described previously [33]. Briefly, rat vibrissa follicles were harvested from male Wistar rats that were 23 days old. To accomplish this, the rats were sacrificed under carbon dioxide (CO_2_). Next, both the left and right mystacial pads were removed from the rats and placed in a 1:1 (v/v) solution of Earle’s balanced salts solution (EBSS; Sigma) and PBS that contained 100 unit/mL of penicillin and 100 μg/mL of streptomycin. Anagen vibrissa follicles were then carefully dissected under a stereomicroscope (Olympus, Tokyo, Japan) from posterior parts of the mystacial pads with considerable care being taken to remove the surrounding connective tissue without damaging the vibrissa follicle. Using this method we were able to routinely isolate more than 40 follicles from each animal. The isolated follicles were then placed in separate wells in 24-well plates that contained 500 μL of Williams medium E (Gibco, Grand Island, NY, USA) supplemented with 2 mM l-glutamine (Gibco, NY, USA), 10 μg/mL insulin (Sigma), 50 nM hydrocortisone (Sigma), 100 unit/mL penicillin and 100 μg/mL streptomycin at 37 °C and cultivated in an atmosphere comprised of 5% CO_2_ and 95% air. The isolated follicles were then treated with vehicle (DMSO diluted 1:1000 in Williams medium E) as a control and *I. sinicola* extract (1, 10 and 100 μg/mL). Minoxidil (Sigma) was used as a positive control in the culture systems [41]. The culture medium was changed every 3 days and photographs of the cultured rat vibrissa follicles were taken using a stereomicroscope for 3 weeks. The length of the hair follicles was measured using a DP controller (Olympus, Tokyo, Japan).

### 4.4. Hair Growth Activity *in Vivo*

Anagen was induced on the back skin of C57BL/6 mice that were in the telogen phase of the cycle by depilation, as described previously [25]. Briefly, 6-week-old female C57BL/6 mice were allowed to adapt to their new environment for one week. The anagen was then induced in the back skin of the 7-week-old female C57BL/6 mice by shaving, which led to synchronized development of anagen hair follicles. From the following day (day 1), 0.2 mL of *I. sinicola* extract in 50% ethanol was topically applied every day for 34 days. 5% Minoxidil (MINOXYL™; Hyundai Pharm. Co. Ltd., Cheonan, Chungnam, Korea) was used as a positive control. The back skin of the mice was then observed and photographed at 1, 12, 19, 26 and 34 days after shaving. For the quantitative assessment, dotmatrix planimetry was performed [42]. 

### 4.5. Assay for Prostatic 5α-Reductase Activity

Male SD rats (8 week old) were sacrificed with CO_2_. The rat prostates were removed from their capsules, washed with saline, and stored at −80 °C. Frozen tissues were thawed on ice and procedures were carried out at 4 °C. The tissues were homogenized with a Polytron homogenizer (Brinkman Instruments, Westbury, NY, USA) in 5–6 tissue volumes of medium A (0.32 M sucrose, 1 mM dithiothreitol (DTT), 0.2 mM phenylmethylsulfonylfluoride (PMSF); and 20 mM potassium phosphate buffer, pH 6.6). The homogenates were centrifuged at 1500 g for 20 min. The pellets were recovered, washed with three tissue volumes of medium A, and centrifuged two additional times at 400 g for 10 min. The washed pellets were suspended in medium A and stored at −80 °C until use. The suspension (2.5 mg protein/mL as determined by the Bradford assay using Bio-Rad reagents) was used as source of 5α-reductase. 5α-reductase activities were analyzed as previously described [43]. The reaction mixture had a final volume of 500 μL and contained 1 mM DTT, 40 mM potassium phosphate buffer, pH 6.6, 2 mM NADPH, and 120 nCi [1,2,6,7-^3^*H*] testosterone. Triplicate reactions were initiated when the reaction mixture was added to the rat prostatic enzyme fraction (250 μg of protein) containing 0.2% DMSO (as a control), *I. sinicola* extract (0.1, 1 and 10 μg/mL), or octaphlorethol A (0.01, 0.1 and 1 μM). Finasteride 2 nM (Merck-Sharpe-Dohme, Granville, NJ, USA) was used as a positive control. The mixture was incubated at 37 °C for 60 min, and then stopped by adding 1 mL of ethyl acetate and mixing for 1 min. After centrifugation at 1000 g for 5 min, the organic phase was removed, dried under a heating plate, dissolved in 50 μL of ethyl acetate containing 500 μg/mL of testosterone and 500 μg/mL of DHT, and applied to a silica gel 60 F254 TLC plate (Merck). The plate was developed in a solvent system consisting of an ethyl acetate:cyclohexane (1:1) solution, and the plate was air dried. Testosterone was visualized under UV light (254 nm) and DHT was detected using a 10% H_2_SO_4_ solution and posteriorly heating the plate. Under these conditions, DHT develops a classical dark yellow color. Areas containing androgen were removed and the strips were soaked in 5 mL of ULTIMA GOLD™ Cocktail (PerkinElmer, Boston, MA, USA) and radioactivity was measured by a liquid scintillation counter (Packard Bioscience, Meriden, CT, USA). The activity of 5α-reductase was expressed as a ratio calculated by the equation: [DHT/(T + DHT)] × 100. 

### 4.6. Assay for the Proliferation of DPCs

Rat vibrissa immortalized dermal papilla cell (DPCs) [44] was donated by the Skin Research Institute, Amore Pacific Corporation R & D Center, Korea. The DPCs were cultured in DMEM (Hyclone Inc., Logan, UT, USA) supplemented with 10% fetal bovine serum (FBS; Gibco, Grand Island, NY, USA) and penicillin/streptomycin (100 unit/mL and 100 μg/mL, respectively) at 37 °C in a humidified atmosphere under 5% CO_2_.

The proliferation of DPCs was evaluated by measuring the metabolic activity using a 3-[4,5-dimethylthiazol-2-yl]-2,5-diphenyltetrazolium bromide (MTT) assay [45]. Briefly, DPCs at 1.0 × 10^4^ cells/mL were seeded into 96-well plate, cultured for 24 h under 1% serum conditions, and then treated with vehicle (DMSO diluted 1:1000 in serum-free DMEM) as a control, the *I. sinicola* extract (0.1, 1 and 10 μg/mL), and octaphlorethol A (0.001, 0.01, 0.1 and 1 μM) for 4 days. After incubation, 0.1 mg (50 μL of a 2 mg/mL solution) of MTT (Sigma) was added to each well, and the cells were then incubated at 37 °C for 4 h. Next, the plates were centrifuged at 1000 rpm for 5 min at room temperature and the media was then carefully aspirated. DMSO 200 μL was then added to each well to dissolve the formazan crystals and the absorbance of the plate at 540 nm was then read immediately on a microplate reader (BioTek Instrument Inc., Winooski, VT, USA). All experiments were performed three times and the mean absorbance values were calculated. The results are expressed as the percentage in the absorbance caused by treatment with *I. sinicola* extract or octaphlorethol A compared to that of the untreated controls. Minoxidil (Sigma) was used as a positive control. 

### 4.7. Western Blot Analysis

The DPCs (1.0 × 10^5^ cells/mL in 100 mm dishes) were pre-incubated for 24 h under 1% serum conditions, and the cells were treated with *I. sinicola* extract (1 and 10 μg/mL) and minoxidil (10 μM) as a positive control for 24 h. The cells were washed twice with ice-cold PBS. The cells were lysed in lysis buffer [50 mM Tris-HCl (pH 7.5), 150 mM NaCl, 2 mM EDTA, 1 mM EGTA, 1 mM NaVO3, 10 mM NaF, 1 mM dithiothreitol (DTT), 1 mM phenylmethylsulfonylfluoride (PMSF), 25 μg/mL aprotinin, 25 μg/mL leupeptin and 1% NP-40] to obtain whole cell protein and kept on ice for 30 min. The cell lysates were centrifuged at 15,000 rpm at 4 °C for 15 min. Supernatants were stored at −20 °C until analysis. Protein concentration was determined by the Bradford method [46]. Equal amounts of protein were separated on 8%–12% Sodium dodecyl sulfate polyacrylamide gel electrophoresis (SDS-PAGE) gels. And then proteins were transferred onto polyvinylidene fluoride (PVDF) membranes (Bio-Rad, Hercules, CA, USA) with a glycine transfer buffer [192 mM glycine, 25 mM Tris-HCl (pH 8.8), 20% MeOH (v/v)] at 100 V for 2 h. After blocking with 5% nonfat dried milk in Tween-20-TBS (T-TBS) (50 mM Tris, pH 7.6, 150 mM NaCl, 0.1% Tween-20), each membrane was incubated with specific primary antibodies against CDK2 (1:2000), Cyclin E (1:2000), p27^kip1^ (1:1000), phospho-GSK3β (1:1000), GSK3β (1:1000), β-catenin (1:2000) and β-actin (1:5000) at 4 °C overnight. The membrane was incubated with a secondary HRP antibody (1:5000) at room temperature for 1 h. The membrane was exposed on X-ray film (AGFA, Mortsel, Belgium), and protein bands were detected using West-zol (Intron, Seoul, Korea). Band intensities were quantified with the NIH Image software.

### 4.8. Statistical Analysis

Results are expressed as the mean ± the standard deviation (S.D.) or standard errors (S.E.) of at least three independent experiments. The Student’s *t*-test was used to determine the statistical significance (*p <* 0.05) of the differences between the values for the various experimental and control groups. 

## 5. Conclusions

In conclusion, the results of this study demonstrated that *I. sinicola* extract and octaphlorethol A are capable of preventing hair loss via the proliferation of DPCs and 5α-reductase inhibition. These findings provide a possibility for the development of octaphlorethol A, a principal of *I. sinicola*, as a therapeutic agent for the treatment of hair loss.
